# Place-Based Policies and Carbon Emission Efficiency: Quasi-Experiment in China’s Old Revolutionary Base Areas

**DOI:** 10.3390/ijerph20032677

**Published:** 2023-02-02

**Authors:** Huwei Wen, Yutong Liu, Yulin Huang

**Affiliations:** 1 School of Economics and Management, Nanchang University, Nanchang 330031, China; 2 Ji Luan Academy, Nanchang University, Nanchang 330031, China

**Keywords:** place-based policies, total-factor carbon emission efficiency, less developed regions, balanced regional development, China

## Abstract

Regional imbalance is a typical feature of economic and social development in China, and place-based policies aimed at promoting balanced regional development may bring challenges to low-carbon goals. This study uses the panel data of China’s prefecture-level cities from 2003 to 2019 to investigate the impact of place-based policies on carbon emission efficiency using a quasi-experimental method. Results indicate that place-based policies significantly reduce the regional total-factor carbon emission efficiency. The difference-in-differences method based on propensity score matching and entropy balancing matching consistently supports the finding that carbon emission efficiency decreases after policy intervention. Place-based policies lead to a significant decline in capital allocation efficiency but have an insignificant impact on labor allocation efficiency. Moreover, place-based policies result in the expansion of carbon-intensive industries but hinder the progress of the financial technology of financial institutions. Nevertheless, place-based policies do not lead to the deterioration of environmental quality. Among the advantages of these policies are the significant promotion of regional digitization and increased fiscal expenditure on science and technology. Political promotion, carbon regulation, trade policies, and other conditional factors may be optimally designed to promote low-carbon development in the old revolutionary areas.

## 1. Introduction

Climate change, which is caused by rising temperature, is a global challenge as humans enter the industrial age, calls for international cooperation and coordination to reduce greenhouse gas emissions [1]. In line with the climate-control targets agreed upon in the Paris Agreement, carbon dioxide emissions must be cut by 45% in the next decade to reach net zero by the middle of the century. However, there are still many less-developed regions and low-income people in the world, and an increase in survivable carbon emissions caused by economic growth is inevitable, bringing numerous challenges to emission reduction. Less-developed regions may also have the advantage of backwardness, and technology spillovers can help these regions achieve low-carbon development. However, the transfer of low-carbon technology is not spontaneous, and there are many constraints [2], thereby also leading to the fact that carbon emissions in developing countries are increasing significantly. Hence, the path of low-carbon development and carbon emission efficiency improvement should be explored in less-developed regions.

China is a country with a large population and geographical area and is characterized by unbalanced regional development. To reduce the imbalance of regional development, China has implemented a series of strategic plans for place-based policies, one of which is the revitalization and development strategy of the old revolutionary base areas [3]. The old revolutionary base areas in China are typically less-developed regions, which are characterized by relatively poor natural conditions, relatively remote geographical locations, and evidently lagging development [4]. The revitalization strategy of the old revolutionary base areas aims to reduce the gap in economic and social development through infrastructure construction, government subsidies, tax incentives, talent transfer, development zone construction, industrial cultivation, and other measures. Although the economic benefits of place-based policies can be considerable, government intervention can also lead to increased carbon emissions and reduced carbon efficiency [5]. In general, balanced regional development may bring pressure on China to promote carbon peaking and carbon neutralization.

Numerous studies have explained the imbalance of regional economic development from the perspectives of system, culture, and geography, and growth theory also provides policy suggestions to narrow the economic gap [6]. Classical economics posits that government intervention in regional development should be place-neutral, and barriers to the flow of labor and other factors should be eliminated. When the marginal output of labor reaches the maximum, the regional imbalance of residents’ living standards will be eliminated. Some advocates of development economics believe that place-based policies are necessary for balanced regional development, and tax incentives and subsidies can enable less-developed regions to obtain certain competitiveness [7]. Some studies have shown that place-based policies violate market competition laws and may have adverse effects in terms of policy traps, crowding out resources, and resource curses, thereby reducing economic efficiency and environmental performance [8]. The revitalization strategy of old revolutionary base areas is a series of place-based policies to promote the economic and social development of less-developed regions in China, and relatively little research has been conducted on its economic and social effects.

Carbon emission efficiency is one of the important indicators in evaluating sustainable development in pursuit of carbon neutrality [1], which also reflects the decarbonization of the regional economy. Place-based policies may have complex effects on regional carbon emission efficiency, which arise from the more advanced capital and technology brought about by these policies and also from the decline in resource efficiency caused by policy intervention [9]. Empirical evidence supports the claim that place-based policies are effective in promoting economic development in less-developed regions, and some studies have found that these policies inhibit regional innovation and bring environmental challenges [10,11]. However, the impact of place-based policies on environmental quality also depends on the regional economic development and environmental quality needs of residents; some regions with better economic development have also achieved a win–win situation after implementing place-based policies [12]. Owing to the low-carbon awareness of residents and the low-carbon constraints of the government, place-based policies targeting less-developed regions may have considerably large adverse effects on carbon emission efficiency.

This study takes the implementation of the revitalization and development strategy of the old revolutionary base areas in China as a quasi-experiment and uses the panel data of 132 prefecture-level cities from 2003 to 2019 to examine the impact of place-based policies on the total-factor carbon emission efficiency. The economic and social characteristics of the old revolutionary base areas are different from those of other areas, so this study adopts the method of propensity score matching (PSM) and entropy balancing matching to overcome the interference of group characteristics on the estimation of policy effect. Results indicate that place-based policies significantly have reduced the total-factor carbon emission efficiency. That is, the low-carbon sustainable development of less-developed regions is one of the major challenges for China to achieve the goal of carbon neutrality. Place-based policies significantly reduced the efficiency of capital factor allocation and increased the proportion of carbon-intensive industries, but did not significantly reduce environmental quality. Trade factors have a significant negative moderating effect on the low-carbon effect of place-based policies.

This research expands the existing literature in the following respects. First, this study focuses on the low-carbon development of the old revolutionary base areas in China. Numerous regions in China are still less developed, and the living carbon emissions in these regions are increasing rapidly. This study also provides insights into the low-carbon development of less-developed regions. Second, this study reveals the relationship between place-based policies and carbon emission efficiency, which has contributed to the theoretical development of regional economics. Specifically, this study uses policy shock as a quasi-experiment to accurately identify causality. The combination of propensity score matching and entropy balance matching, and difference-in-difference (DID) regression effectively overcomes the interference caused by the economic and social characteristics of the old revolutionary base areas. Lastly, this study helps optimize policies for balanced regional development, particularly in promoting cooperation in low-carbon development among regions. Regional imbalances are not limited to being within an economy, but also between different countries, which pose challenges for global carbon reduction, and this study also provides some insights into international cooperation for climate governance.

## 2. Background and Theoretical Analysis

### 2.1. Background

The old revolutionary base areas in China refer to the revolutionary base areas created under the leadership of the Communist Party of China during the Agrarian Revolutionary War and the War of Resistance Against Japan. These areas are distributed in over 1300 counties (cities, districts) in 28 provinces. The old revolutionary base areas are less developed areas mainly in mountainous areas affected by natural, traffic, historical, cultural, and other factors. Owing to relatively slow development, insufficient self-development capacity, prominent poverty problems, and other reasons, economic development is slow and regional economic development is uneven. To promote the development of the old revolutionary base areas, China implements government intervention and strategic planning for the revitalization and development of the old revolutionary base areas [13], which is generally divided into four stages.

First stage: “relief” policy (1978–1986). The Chinese government stipulated that the income tax of agricultural, industrial, and commercial economic entities in the old revolutionary base areas should be exempted. Moreover, the old revolutionary base areas office should be set up to be responsible for the guidance of the old revolutionary base areas. The Ministry of Finance also established a special fund to support the development of economically underdeveloped areas. Second stage: “open” policy (1986–2001). In response to the unbalanced development of the eastern and western regions, plains, and mountains, the state issued numerous targeted poverty reduction policies to solve the problems of food and clothing for 80 million people in seven years. Third stage: “participatory” policy (2001–2012). The state issued policies again to define the basic principles and objectives of poverty alleviation and development in the next decade, mainly through the “whole village promotion” mode, to activate the development of old revolutionary base areas by means of transfer payments and project support, among others. Fourth stage: “precision” policy (since 2012). The state implements differentiated support policies for key old revolutionary base areas and formulates policies in accordance with local conditions for the five key old revolutionary base areas: the former Central Soviet Area (e.g., Gannan); the old revolutionary base areas in Shaanxi, Gansu, and Ningxia; the old revolutionary base areas in Zuojiang and Dabie Mountains; and the old revolutionary base areas in Sichuan and Shaanxi.

Promoting the revitalization and development of the old revolutionary base areas is a practical action to consolidate and expand the achievements of poverty alleviation in the new development stage, and also an urgent need to promote regional coordinated development in the new era. This undertaking is an important link to gradually achieve common prosperity and start a new journey of socialist modernization. In 2012, the State Council issued several opinions on supporting the revitalization and development of the former Central Soviet Areas, such as Gannan; approved the implementation of the revitalization and development plans of the old revolutionary base areas, such as Shaanxi, Gansu, Ningxia, Zuojiang, Dabie Mountains, Sichuan, and Shaanxi; and deployed and implemented several support measures and major projects. It covers a series of national support policies, such as financial fund support and preferential tax, counterpart support, industrial park construction, science and technology education, talent, land, and ecological compensation policies.

### 2.2. Literature and Theoretical Analysis

Regional unbalanced development is one of the basic characteristics of China’s economy, and regional balance policies are widely formulated and used [14]. The country has successively implemented regional coordination strategies, such as the western development and revitalization of the old industrial base in the northeast, to alleviate the imbalance of regional development. Some studies believe that regional coordination strategy plays an important role in the economic and social development of underdeveloped regions by promoting improvement in the infrastructure and industrial development in less-developed regions through preferential policies and policy support [15,16]. Moreover, some studies have held that regional coordination strategy violates the law of complete market competition, which may be unable to improve the pattern of unbalanced economic development among regions [8].

Place-based policies aim to encourage the development of specific regions to achieve balanced spatial growth and reduce the imbalance in regional development [17]. In particular, place-based policies may have different purposes, including eliminating poverty, promoting industrial clusters, and encouraging innovation resource agglomeration. For example, place-based policies of establishing high-tech zones in China can encourage regional innovation and promote entrepreneurial activities [18]. Some studies have found that China’s place-based policies have had a positive impact on investment, employment, productivity, and wages [19]. Nevertheless, these policies may violate the market law, which may result in a series of undesirable results, including a decline in the efficiency of resource allocation and an increase in environmental pollution. Some pieces of empirical evidence have shown that place-based policies aimed at promoting industrial economic development have caused considerably serious environmental pollution [20,21]. Therefore, if environmental issues are excluded from the framework of local policies, then local governments have the motivation to reduce environmental regulations to achieve the direct goal of policy design [22], thereby bringing challenges to sustainable development.

The revitalization and development of the old revolutionary base areas is an important component of China’s regional coordinated strategy and even constitutes one of the key weaknesses of regional coordinated development. The old revolutionary base areas still face many problems in economic and social development. In recent years, a consensus has indicated that the state prioritizes supporting the revitalization and development of the old revolutionary base areas. As a less-developed region, the old revolutionary base areas have received a series of policy support. This undertaking is an important choice to solve China’s increasingly serious regional development imbalance and also an objective compensation demand based on the huge sacrifice made by the old revolutionary base areas to support the cause of founding a nation. Place-based policies of the old revolutionary base areas tend to transfer government financial transfer payments, subsidies, financial services, laws and regulations, and other resources to underdeveloped areas [23]. An increase in government intervention tends to promote economic growth, accelerate industrial transformation, and improve people’s livelihood, which may lead to the formation of zombie enterprises and distort resource allocation [11]. Most of the old revolutionary bases in China are in the less-developed stage of economic development. The urgent task of policy intervention is to improve the disposable income and quality of life of residents. Given that carbon emissions are not directly related to the well-being of residents, place-based policies of the old revolutionary base areas may disregard low-carbon development and the improvement in carbon-emission efficiency. Therefore, this paper proposes the following hypothesis to be tested.

**Hypothesis.**  *The implementation of place-based policies to revitalize China’s old revolutionary base areas reduces the total-factor carbon emission efficiency.*

## 3. Sample and Research Design

### 3.1. Sample and Data

This study selects 132 prefecture-level cities in the provinces involved in the revitalization and development plan of five key old revolutionary base areas from 2003 to 2019 as research samples, with 2244 observations. According to the revitalization and development plan of the five key old revolutionary base areas, this study takes prefecture-level cities within the planning scope as the treatment group and areas excluded in the planning scope in the planning provinces as the control group. The treatment group included 33 prefecture-level cities, while the control group included 99 prefecture-level cities. Data were mainly from the China Urban Statistical Yearbook, and some variables were collected and collated by the author.

### 3.2. Empirical Model

This model defines the interaction term between the city group dummy variable (ORBArea_i_) and dummy variable before and after the implementation of the policy (Post_t_). Thereafter, the difference-in-differences model is used to evaluate the effect of place-based policies on carbon emission efficiency. The empirical model is set as follows:

(1) $$TFCEE_{it}=\alpha_{0}+\delta \cdot ORBArea_{i}\times Post_{t}+\sum_{k=1}^{K}\gamma_{k}Controls_{kit}+\mu_{i}+\lambda_{t}+\varepsilon_{it},$$

where TFCEE_it_ indicates the total-factor carbon emission efficiency of region i in period t and Controls_kit_ represents the kth control variable. The individual fixed effect (μ_i_) and year-level fixed effect (*λ_t_*) are also controlled. The coefficient of the interaction term (δ) is the main estimator of the study. If it is significantly less than zero, then it indicates that place-based policies aim to significantly reduce carbon emission efficiency. The econometric software Stata 16.0 is used to estimate the parameters of the model.

### 3.3. Variable Definition and Description

The dependent variable is total-factor carbon emission efficiency (TFCEE). Carbon emission efficiency refers to the potential saving degree of carbon emissions after considering the input and output of the decision-making unit. Moreover, the epsilon-based measure (EBM) model and slacks-based measure (SBM) model are used to calculate carbon emission efficiency [24,25]. The definitions of the main variables involved in this study are shown in Table 1. Input and output data are required to calculate carbon emission efficiency. Input data include capital, labor, expected output data (gross domestic product (GDP)), and unexpected output data (carbon emissions). The software of MATLAB is used to run the SBM model and the EBM model.

The core explanatory variable is the place-based policies of the old revolutionary base areas. This study uses the quasi-experimental event impact as the proxy variable. In particular, the interaction term of the group dummy variable (ORBArea) and year dummy variable (Post) serves as the core explanatory variables. The dummy variable of ORBArea is equal to 1 for regions in the revolutionary base area; otherwise, it is equal to 0. The year dummy variable of Post takes a value of 1 after 2012 and 0 before 2012.

A series of factors affecting carbon efficiency have been controlled. Regional economic development (lnRGDP) is measured by the logarithm of per capita GDP. Government governance (Gover) reflects the ability of local governments to govern the economy, which is measured by the proportion of fiscal expenditure in GDP. Industrial structure (Jndustry) is defined as the proportion of manufacturing employment within total employment. Foreign direct investment (FDI) has technology spillovers or low-end lock-in, which is measured by the proportion of foreign direct investment in the total output [26]. Environmental regulation (ER) affects low-carbon technological progress, which is defined as the proportion of the employees in the environmental industry in total employment [27]. Population density (PDensity) reflects the endowment of labor resources, which is measured by the ratio of the permanent population to the land area. Human capital (Human) is measured by the average wages of regional employees. If there is no factor price distortion, then wage difference should reflect human capital. The number of primary and secondary school students (Student) is measured by the number of primary and secondary school students in the resident population. Digitalization (Digital) is the logarithm of employees in the information technology service industry. Lastly, independent innovation (lnPatent) is measured by the logarithm of the number of regional patents.

Table 2 shows the descriptive statistics of the main variables in this study. The correlation coefficient of carbon emission efficiency obtained by the two measurement methods is 0.3958, which is relatively different. In addition, the correlation coefficient between the DID term and the two dependent variables is a negative correlation, indicating that the place-based policies may reduce the carbon emission efficiency of the old revolutionary base areas. These control and dependent variables have a certain correlation, which is reasonable to include in the model.

## 4. Research Results and Analysis

### 4.1. Benchmark Results and Analysis

Table 3 shows the impact of intervention policies in the old revolutionary base areas on regional carbon emission efficiency. The first three columns use the carbon emission efficiency calculated based on the SBM model as the dependent variable. The last three columns use the carbon emission effect calculated based on the EBM model as the dependent variable. Columns (3) and (6) are the benchmark results of this study.

Results indicates that the policy intervention has a significant effect on reducing carbon emission efficiency. That is, place-based policies have brought challenges to low-carbon development. Except for column (3), coefficients of ORBArea × Post in Table 3 are significantly negative. Compared with the average carbon emission efficiency, the policy intervention resulted in an average decrease of 7.45% in carbon emission efficiency, which is of immense economic significance. This result is consistent with theoretical expectations. Most of the old revolutionary base areas in China are in the less-developed stage of economic development, and the urgent task of policy intervention is to improve the disposable income and quality of life of residents. Therefore, place-based policies encourage substantial industrial investment and economic expansion, resulting in an increase in energy consumption and carbon emissions.

Economic development can provide additional available resources for low-carbon development, which shows a significant positive impact on carbon emission efficiency. Government intervention (Gover) would reduce the efficiency of resource use, including carbon, thereby reducing the efficiency of carbon emissions. Foreign direct investment conforms to the pollution paradise hypothesis or shows the lock-in effect of high carbon links, which has a negative impact on carbon emission efficiency [28]. The development of the manufacturing industry has not reduced the carbon emission efficiency, which is mainly due to the higher production efficiency of the manufacturing industry. Digitalization also contributes to low-carbon development and has a positive impact on carbon emission efficiency [29]. The number of primary and secondary school students reflects residents’ willingness to settle down; the higher the value, the more likely it is to favor the economic model of sustainable development. Accordingly, the number of primary and secondary school students is conducive to improving carbon emission efficiency. Technological progress is not necessarily conducive to low-carbon development, and a large number of technologies in the industrial sector are increasing energy consumption and carbon emissions [30]. Hence, technological innovation in the old revolutionary base areas is not conducive to low-carbon development.

### 4.2. Regression Results Based on Matching Samples

The characteristics of various economic and social variables in the old revolutionary base areas are relatively different from those in other regions. The nonlinear effects of these characteristics are difficult to capture in the regression model, thereby possibly leading to a potential deviation in the DID estimation results. Therefore, this part adopts a series of matching methods to reduce the difference in characteristic variables between the two groups of regions. We specifically use propensity score matching and entropy balancing matching and utilize the matched subsamples thereafter to conduct DID regression analysis. On the bases of key characteristics of the old revolutionary base areas, eight variables are selected as matching variables in this study, four of which are the influencing factors of carbon emissions. The other four are grain output per capita (lnFood), medical conditions (Medical), the ratio of primary and secondary school students to teachers (Education), and financial resource flow (Finance). Medical conditions are measured by the number of beds per thousand people, while the flow of financial resources is measured by the ratio of the deposit balance of financial institutions to their loan balance.

Table 4 shows the regression results of the Probit model with respect to whether or not various regions can become old revolutionary base areas. Note that medical resources and population density significantly affect its probability, thereby also reflecting the huge differences between the old revolutionary base areas and other regions in terms of medical care and population concentration, among others. Although other variables are not significant, they also have some explanatory power. These coefficients are not significant, which is also consistent with reality. The selection of old revolutionary base areas is determined by historical political factors and not by current economic and social variables. The pseudo R-squared of the model can also further explain why the selection of old revolutionary base areas is exogenous. Nevertheless, this study uses propensity score matching not to overcome the endogeneity of the selection of old revolutionary base areas but to overcome the bias of estimation results caused by the huge differences of the related variables in different regions and their nonlinear relationships.

**Figure 1:**
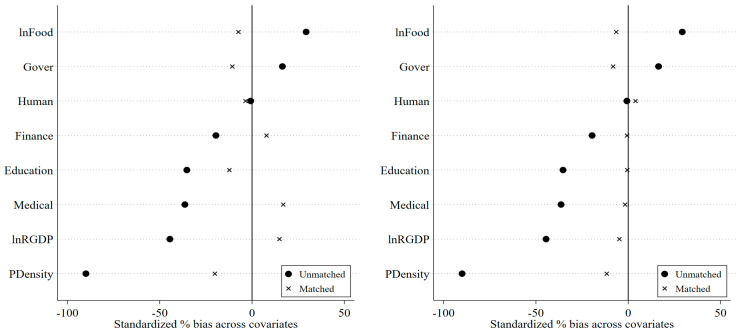
Balance test of covariates before and after propensity score matching. Note: The figure was drawn using Stata 16.0.

Figure 1 shows the balance test of covariates for propensity score matching. The left figure is the nearest neighbor distance matching of 1:2, and the right figure is the result of kernel matching. Figure 1 also shows that there are some differences in the economic and social characteristics of the old revolutionary base areas relative to other areas. Most of the old revolutionary base areas are mountainous areas with complex terrains; thus, these regions are characterized by low population density, low economic development, and inadequate medical, educational, and financial resources. It is necessary to evaluate the actual effect of the policy using a matched sample. Due to the uniqueness of the economic and social characteristics of the old revolutionary base areas, this study mainly uses the samples obtained from PSM and entropy balancing matching to evaluate the economic environmental effects of the place-based policies in the old revolutionary base areas.

Note that the group differences in covariates before matching are large, while the differences in covariates after using propensity score matching are substantially reduced. In particular, the deviation of covariates is less than the critical value of 20%, indicating that the matched samples have passed the balance test. Neighborhood matching and kernel matching can significantly reduce the difference in group features. Therefore, this study obtained two groups of subsamples through propensity score matching. Before the implementation of the policy, there was no systematic group difference between the two groups of subsamples.

This study uses entropy balancing to keep the covariate characteristics of the control and treatment groups as consistent as possible. Entropy balancing matching aims to match the samples of the treatment and control groups to the greatest extent and considers the multi-dimensional balance of covariates, such as the first, second, and higher moments [31]. Table 5 shows the moment characteristics of covariates of the two groups of samples before and after matching entropy balance. The first three columns are the mean, variance, and skewness of the relevant variables for the sample in the treatment group, followed by the corresponding statistics for the unmatched sample of control group. The last three columns are the corresponding statistics for the matched sample in the control group. These results show that the moment characteristics of the control group covariates and those of the treatment group are close to each other.

Table 6 shows the results of the DID regression analysis using subsamples obtained by the three matching methods, which maximally avoided differences in group characteristics between policy interventions. The first two columns are the results of regression based on the neighboring matched subsamples, the next two columns are the results of regression based on kernel matching, and the last two columns are the results of regression based on entropy balancing matching. These regressions based on kernel matching and entropy balance matching are weighted regressions based on the weights obtained by matching. Thus, the observed values used for columns (3) to (6) are close to the observed values for the full sample.

The results in Table 6 consistently show that place-based policies significantly reduce total-factor carbon emission efficiency. In column (1), the coefficient of ORBArea × Post is negative but not significant, with a T-value of 1.28. This generally supports the notion that policy intervention has a negative impact on total-factor carbon emission efficiency with a high probability. In column (2), the DID coefficient is significantly negative at the 5% level, and the two columns illustrate the negative impact of place-based policies on carbon efficiency. In columns (3) to (6), the coefficients of ORBArea × Post are significantly negative at the 5% level, demonstrating a significant reduction in carbon emission efficiency. There may be some differences in the magnitude of the coefficients, and different matching methods or different measures of carbon efficiency do not change the results of the reduction in carbon efficiency caused by policy interventions. Empirical evidence shows that the hypothesis of this study is valid, and the place-based policies of revitalizing old revolutionary base areas in China have a negative effect on carbon efficiency.

### 4.3. Parallel Trend Test for the DID Regression Analysis

**Figure 2:**
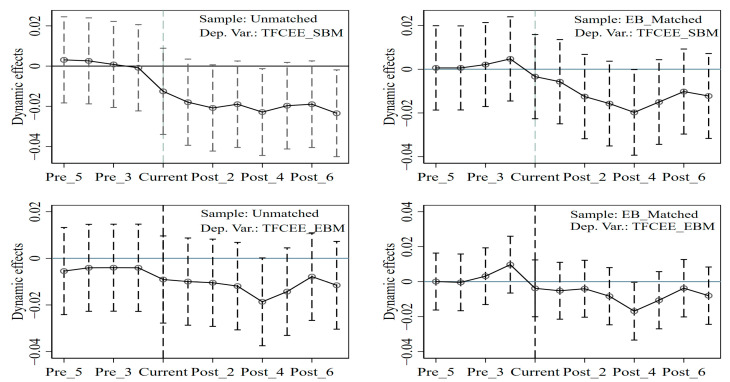
Results of the parallel trend test for the DID regression. Note: The figure is drawn using Stata 16.0.

The precondition of using the DID method to evaluate the effect of policy intervention is that the target variable satisfies the assumption of parallel trend before policy intervention. Figure 2 shows the results of the parallel trend test, in which two subgraphs are the parallel trend test for the full sample and the other two subgraphs are the parallel trend test for the entropy-balancing matching subsample. Moreover, there are no significant policy effects before policy intervention in either the full sample or the matched sample. That is, the parallel trend assumption is satisfied between the treatment and control groups. After police intervention, policy effects are negative and are significant in some years, showing that the policy intervention of revitalizing old revolutionary base areas reduces the total-factor carbon emission efficiency.

### 4.4. Empirical Results of the Mechanism Analysis

Place-based policies aim to help specific regions achieve economic growth through a series of government intervention measures. One of the potential challenges of government intervention is that it may reduce the efficiency of resource allocation [32,33]. In this study, the ratio of the factor marginal output of a region to the factor marginal output at the national level is taken as the proxy indicator of factor allocation efficiency. The higher the value of the ratio indicator, the lower the efficiency of the factor allocation. This study specifically defines the allocation efficiency indicators of capital and labor factors. Table 7 shows the impact of policy intervention in the revitalization of old revolutionary base areas on the allocation efficiency of capital and labor.

The results in Table 7 show that place-based policies of the old revolutionary base areas significantly reduced the allocation efficiency of capital elements but have an insignificant impact on the efficiency of labor allocation. Although the coefficients of ORBArea × Post are negative, only the coefficients in columns (1) and (2) are significant. That is, the evidence proves that the efficiency of capital allocation is reduced owing to policy intervention. Less-developed characteristics of the old revolutionary base areas show that they have relatively rich labor endowment, and place-based policies of the old revolutionary base areas enable the labor force in these areas to be used more effectively, which would not adversely affect the efficiency of labor allocation. By contrast, policy intervention may lead to the transfer of capital to these regions in violation of market rules, resulting in a smaller marginal output of capital and lower efficiency of capital allocation. The analysis based on the efficiency of resource allocation shows that place-based policies of the old revolutionary base areas may face the challenge of long-term sustainable development. Although these policies have brought immense economic and social achievements, the key to long-term sustainable development is to improve the endogenous power of the economy.

Place-based policies of the old revolutionary base areas may also lead to changes in the industrial structure, which would have an impact on carbon emission efficiency. This study mainly examines the industrial structure effect of policy intervention from the perspectives of energy-intensive industries and science and technology finance. Table 8 shows the empirical results of the industrial structure analysis. Columns (1) and (2) show the impact of place-based policies on the proportion of energy-intensive industries, which is measured by the logarithm of the number of employees in energy-intensive sectors. Columns (3) and (4) show the development of science and technology finance, which is measured by the number of media reports on science and technology finance business by banks in the region. Financial institutions play an important public function in place-based policies, thereby hindering the development of financial institutions, particularly the corresponding finance for science and technology.

The results in Table 8 show that the intervention policies in the old revolutionary base areas significantly increased the proportion of energy-intensive sectors and significantly hindered the development of regional science and technology finance. In columns (1) and (2), the coefficients of ORBArea × Post are significantly positive at the 1% level, implying that the place-based policies of the old revolutionary base areas significantly increased regional energy demand. The increase in energy demand and proportion of energy-intensive industries would eventually lead to an increase in carbon emissions and a decline in carbon emission efficiency. In columns (3) and (4), coefficients of ORBArea × Post are significantly negative at the 1% level. That is, policy intervention hinders the development of finance for science and technology. Given that finance for science and technology plays an important role in promoting economic efficiency and low-carbon economic development [34], our findings support the mechanism of place-based policies to curb carbon emission efficiency through science and technology finance.

### 4.5. Discussion on the Innovation and Pollution Effects

Innovation is the key path to achieving low-carbon sustainable development. The key to whether or not the place-based policies of the old revolutionary base areas have low-carbon development potential is whether or not they can produce innovative effects [11]. This study defines three proxy variables to measure innovation, namely, logarithm of patents (lnPatent), logarithm of scientific and technological financial expenditure (Tech_Fiscal), and logarithm of the number of employees in the information and communication technology service industry (Digital), to measure independent innovation, technological improvement, and digital innovation, respectively.

Note that the place-based policies of the old revolutionary base areas have an insignificant impact on regional independent innovation but have a significant positive impact on technological improvement and digital innovation. The dependent variable of columns (1) and (2) is independent innovation, while the coefficients of ORBArea × Post are insignificant. Tax incentives or subsidies cannot improve the ability of regional independent innovation, while less-developed regions rely mainly on technology learning and absorption to obtain technological progress. Hence, regional independent innovation has not been improved owing to policy intervention. Fiscal expenditure on science and technology represents the fiscal support of local governments for regional technological progress, which can reflect the situation of regional technological progress. The results in Table 9 show that policy intervention can help these regions obtain technological progress. In addition, policy intervention has also encouraged the increase in ICT employment, indicating that these regions achieved revitalization through digital innovation. Digital innovation offers opportunities in terms of technological pathways for less-developed regions [35].

The place-based policies of the old revolutionary base areas have significantly reduced carbon emission efficiency. Thus, do these policies also lead to a decline in environmental quality indicators? This study selected three proxy indicators of environmental pollution to examine the environmental effects of the revitalization policy of the old revolutionary base areas. These indicators are the air PM2.5 concentration (PM2_5), industrial wastewater discharge (Pollution_Water), and industrial waste gas discharge (Pollution_Gas). The estimated results of the environmental effects of policy intervention are shown in Table 10.

Results indicate that place-based policies did not exacerbate the environmental quality of the old revolutionary base areas in China. The coefficients of ORBArea × Post in columns (1) to (4) are negative but not significant, indicating that place-based policies may improve the environmental quality. For the dependent variable industrial waste gas, the signs of the coefficients of ORBArea × Post are inconsistent and not significant, indicating that the impact of place-based policies is uncertain. Although carbon emission is a type of generalized pollutant, it is different from traditional pollution. Residents are concerned with the discharge of general polluted gas and wastewater, which will have a direct impact on the health and well-being of residents. Therefore, the place-based policies of the old revolutionary base areas would focus on traditional environmental problems, thereby not deteriorating the environmental quality.

### 4.6. Moderating Effect of the Trade Factors

One of the tasks of location-oriented policies is to promote regional industrial development, and thus specific measures are taken to enhance the integration of target regions into the industrial division of labor. Table 11 examines the moderating role of factors such as domestic trade and international trade in the revitalization policy of old revolutionary base areas. Domestic trade (Trade_Domestic) is measured by the ratio of total goods in wholesale and retail trade to GDP, and international trade (Trade_Foreign) is measured by import and export trade to total GDP.

The results show that although there is an insignificant effect of trade factors on carbon emission efficiency, and both international and domestic trade show significant moderating effects. The low-carbon development of China’s old revolutionary base areas is influenced by the changing trade environment. For example, China is actively signing trade agreements with neighboring countries and countries along the Belt and Road, which has a positive effect on promoting the economic development of less-developed regions, but may also bring adverse effects on carbon emission efficiency [36]. Market-based mechanisms need to be designed to encourage economic agents in old revolutionary base areas to take the initiative to adopt low-carbon production technologies.

## 5. Conclusions

Although China has proposed ambitious carbon-neutral goals, the imbalance of its regional economic and social development has also brought many challenges to China’s pursuit of carbon-neutral goals. This study takes the revitalization and development of China’s old revolutionary base areas as the analysis object to investigate carbon emission consumption and the decline of carbon emission efficiency necessary for the economic and social development of China’s underdeveloped regions. By using quasi-natural experimental methods and panel data from 132 prefecture-level cities, this study examined the impact of the place-based policies in old revolutionary base areas on total-factor carbon emission efficiency.

The empirical results show that carbon emission efficiency of the old revolutionary base areas decreased significantly after the intervention of place-based policies. That is, the place-based policies of the old revolutionary base areas are not conducive to low-carbon development. This study also used propensity score matching and entropy balance matching to substantially reduce the differences in the characteristics of covariates between the treatment and control groups. After overcoming the differences in the characteristics of covariates, the results of the DID model consistently support the conclusion that place-based policies reduce the carbon emission efficiency of old revolutionary base areas. A series of causal identification tests in this study show that the place-based policies of the revitalization of old revolutionary base areas brought challenges to low-carbon development.

This study explains the adverse carbon-emission efficiency effect of place-based policies from the perspective of resource allocation efficiency and changes in industrial structure. On the one hand, place-based policies led to a significant decline in the efficiency of resource allocation, particularly capital allocation. On the other hand, place-based policies also resulted in the expansion of carbon-intensive industries and hindered the financial technology progress of financial institutions. Given that policy intervention leads to the decline of resource allocation efficiency and a change in industrial structure that is not conducive to low-carbon development, the decline in carbon emission efficiency under policy intervention can also be understood. This study further examined the innovation and environmental effects of place-based policies. Fortunately, the place-based policies of the old revolutionary base areas did not cause environmental quality deterioration and promoted regional technological progress and digital innovation, but they have an insignificant impact on independent innovation. The industrial division of labor affects the low-carbon effect of place-based policies, and international and domestic trade show significant negative moderating effects.

Our findings in this study provide numerous insights into China’s regional balanced development and global climate governance. First, economic development demands of less developed regions and the growth of carbon emissions for survival are major challenges to global climate governance. Less developed regions may lack motivation and capacity in global climate governance. International organizations should take measures to encourage and help underdeveloped regions to achieve low-carbon development, such as financial support and technology transfer [37]. Second, unbalanced regional development could bring many difficulties to the promotion of the process in China’s carbon neutrality. The Chinese government needs to coordinate and optimize low carbon and regional balance policies to reduce conflict between policies. Third, China has the potential to promote low-carbon development in old revolutionary base areas. Place-based policies did not cause environmental pollution, but they stem from the local government’s concern for environmental issues. If low-carbon development is included in the plan for the revitalization of the old revolutionary base areas, then the old revolutionary base areas are capable of achieving low-carbon development. Lastly, cross-regional and cross-subject cooperation of low-carbon technologies are prerequisites for achieving global climate governance. Market-based mechanisms should be designed to promote low-carbon technological cooperation and increase the motivation of various actors to participate in low-carbon development [38]. In sum, place-based policies can be optimized to achieve the goal of low-carbon development.

This study enriches the theory of balanced regional development from the perspective of a low-carbon economy and also provides theoretical insights for global climate governance under the conditions of unbalanced north–south development. Limitations can be further studied and expanded. Firstly, most of the old revolutionary base areas in China are in the areas between provinces adjacent to borders, and if data on carbon emissions and the related variables are available at the county level, this could allow analysis of the low-carbon development effects caused by administrative borders. Secondly, factors such as political promotion, carbon regulation and trade policies are conditions for our findings, and extended research can examine how these factors act on the low-carbon effects of place-based policies. Thirdly, the old revolutionary base areas are abundant in carbon sink resources such as forests; however, place-based policies may not only directly encroach on forests but also may influence residents’ behavior through income effects, thus causing deforestation. Hence, it is meaningful to investigate the changes in forest carbon sinks in old revolutionary base areas.

## Figures and Tables

**Table 1:** Variable definitions.

Variables	Observations
TFCEE-SBM	Total-factor carbon emission efficiency: calculated by the slacks-based measure model
TFCEE-EBM	Total-factor carbon emission efficiency: calculated by the epsilon-based measure model
ORBArea	Dummy variable: equal to 1 for regions in the old revolutionary base area
lnRGDP	Economic development: logarithm of per capita GDP
Gover	Government governance: measured by the proportion of fiscal expenditure in GDP
Jndustry	Industrial structure: proportion of manufacturing employment in the total employment
FDI	Foreign direct investment: proportion of foreign direct investment in the total output
ER	Environmental regulation: proportion of employees in the environmental industry
PDensity	Population density: measured by the ratio of permanent population to land area
Human	Human capital: measured by the logarithm of the average wages of employees
Student	Students: ratio of primary and secondary school population to the total population
Digital	Digitalization: logarithm of employees in the information technology service industry
lnPatent	Independent innovation: measured by the logarithm of the number of regional patents

Source: Defined by the authors.

**Table 2:** Descriptive statistics.

Variables	Observations	Mean	Std. Dev.	Minimum	Maximum	Corr1	Corr2
TFCEE-SBM	2244	0.3075	0.0952	0.1454	1.0000	1.0000	-
TFCEE-EBM	2244	0.3958	0.1066	0.0866	1.0000	0.3846	1.0000
ORBArea × Post	2244	0.1176	0.3223	0.0000	1.0000	−0.1093	−0.0103
lnRGDP	2244	9.9713	0.7981	4.5951	12.5793	0.1315	0.4945
Gover	2244	0.0593	0.0238	0.0107	0.2380	−0.0207	0.1437
Jndustry	2244	0.4312	0.1351	0.0446	0.8343	0.2329	0.3108
FDI	2244	0.0173	0.0194	0.0000	0.2093	−0.0608	0.1064
ER	2244	0.1371	0.0934	0.0040	1.0190	0.2128	−0.0886
PDensity	2244	0.4100	0.2730	0.0050	1.3400	−0.0398	0.3489
Human	2244	10.3088	0.6246	8.5088	11.8284	0.0043	0.3765
Student	2244	0.1382	0.0445	0.0332	0.5174	0.1684	0.0502
Digital	2244	0.2493	0.1476	0.0100	1.2556	−0.1512	0.4416
lnPatent	2244	4.6480	1.9377	0.0000	10.3496	−0.1269	0.4666

Note: Corr1 and Corr2 represent the correlation coefficients of each control variable and two dependent variables, respectively. The data were calculated by the authors using Stata 16.0.

**Table 3:** Empirical results of DID estimates of policy effects.

Variables	TFCEE-SBM			TFCEE-EBM		
	(1)	(2)	(3)	(4)	(5)	(6)
ORBArea × Post	−0.0217 ***	−0.0237 ***	−0.0229 ***	−0.0064	−0.0280 ***	−0.0112 **
	(0.0052)	(0.0049)	(0.0050)	(0.0045)	(0.0051)	(0.0044)
lnRGDP	0.0190 ***	0.0201 ***	0.0240 ***	0.0284 ***	0.0800 ***	0.0353 ***
	(0.0067)	(0.0058)	(0.0066)	(0.0058)	(0.0061)	(0.0057)
Gover		−0.2923 ***	−0.2458 ***		−0.0351	−0.1040
		(0.0824)	(0.0865)		(0.0859)	(0.0755)
Industry		0.0985 ***	0.1044 ***		0.0621 ***	0.0674 ***
		(0.0199)	(0.0204)		(0.0208)	(0.0178)
FDI		−0.6214 ***	−0.6597 ***		−0.9806 ***	−0.8990 ***
		(0.0910)	(0.0909)		(0.0949)	(0.0793)
ER		−0.1365 ***	−0.1354 ***		0.0363	0.0018
		(0.0244)	(0.0243)		(0.0254)	(0.0212)
PDensity		0.1744 ***	0.1683 ***		0.0913	−0.1023 *
		(0.0597)	(0.0599)		(0.0622)	(0.0523)
Human		−0.0048	0.0190 *		0.0174 **	0.0067
		(0.0070)	(0.0100)		(0.0073)	(0.0087)
Student		0.3857 ***	0.4250 ***		0.0521	0.2692 ***
		(0.0546)	(0.0555)		(0.0569)	(0.0484)
Digital		0.1017 ***	0.1128 ***		0.0387 ***	0.0603 ***
		(0.0128)	(0.0129)		(0.0134)	(0.0112)
lnPatent		−0.0063 ***	0.0001		−0.0141 ***	−0.0014
		(0.0017)	(0.0019)		(0.0018)	(0.0017)
City fixed effect	Yes	Yes	Yes	Yes	Yes	Yes
Year fixed effect	Yes	No	Yes	Yes	No	Yes
R-squared	0.0522	0.1369	0.1657	0.5835	0.4405	0.6214
Observations	2244	2244	2244	2244	2244	2244

Note: Numbers in brackets are standard errors. Asterisks indicate the significance of the corresponding levels: *** (1%), ** (5%), and * (10%). The data were calculated by the authors using Stata 16.0.

**Table 4:** Probit regression results of propensity score matching.

Variables	Coefficients	Std. Err.	z	*p* > z
lnRGDP	−0.1661	0.4125	−0.40	0.6870
lnFood	0.4676	0.3537	1.32	0.1860
Medical	−0.0429	0.0228	−1.88	0.0600
Education	−0.6363	1.0952	−0.58	0.5610
Gover	2.1127	7.4970	0.28	0.7780
Finance	−1.5932	1.1591	−1.37	0.1690
PDensity	−2.8115	0.8828	−3.18	0.0010
Human	1.0750	1.1230	0.96	0.3380
Constants	−4.8990	13.4515	−0.36	0.7160
Number of observations = 132, Pseudo R^2^ = 0.2025				

Note: Regression of sample matching based on 2011 data. The data were calculated by the authors using Stata 16.0.

**Table 5:** Comparison of covariates before and after entropy balancing matching.

Variables	Treat			Control-Unmatched			Control-Matched		
	Mean	Variance	Skewness	Mean	Variance	Skewness	Mean	Variance	Skewness
lnRGDP	10.01	0.2442	0.8726	10.22	0.2019	0.2350	10.01	0.1505	−0.9054
lnFood	−0.91	0.1409	−0.0692	−1.08	0.5202	−2.5220	−0.91	0.1710	0.2470
Medical	29.16	60.7400	0.7281	33.42	212.9000	2.9980	29.18	38.0700	0.8128
Education	2.76	0.0359	0.1289	2.82	0.0230	−0.1031	2.76	0.0195	−0.0932
Gover	0.06	0.0004	0.5292	0.06	0.0003	0.9398	0.06	0.0005	0.8917
Finance	0.54	0.0313	0.9800	0.57	0.0177	0.9576	0.54	0.0085	0.3703
PDensity	0.26	0.0273	0.8950	0.47	0.0824	0.5436	0.26	0.0205	1.1260
Human	10.39	0.0276	0.1720	10.39	0.0293	0.8241	10.39	0.0180	−0.0510

Note: The data were calculated by the authors using Stata 16.0.

**Table 6:** Empirical results of the DID regression based on the matched sample.

Variables	PSM-Neighboring		PSM-Kernel		Entropy Balancing	
	(1) SBM	(2) EBM	(3) SBM	(4) EBM	(5) SBM	(6) EBM
ORBArea × Post	−0.0102	−0.0148 **	−0.0143 ***	−0.0091 **	−0.0150 ***	−0.0106 ***
	(0.0080)	(0.0062)	(0.0050)	(0.0041)	(0.0046)	(0.0039)
lnRGDP	0.0341 ***	0.0473 ***	0.0360 ***	0.0488 ***	0.0320 ***	0.0515 ***
	(0.0105)	(0.0081)	(0.0064)	(0.0053)	(0.0061)	(0.0052)
Gover	−0.2486 *	−0.1420	−0.3528 ***	−0.2096 ***	−0.2964 ***	−0.2488 ***
	(0.1397)	(0.1078)	(0.0972)	(0.0799)	(0.0928)	(0.0783)
Industry	0.1094 ***	0.0780 **	0.0772 ***	0.0813 ***	0.0549 **	0.0658 ***
	(0.0407)	(0.0314)	(0.0244)	(0.0201)	(0.0214)	(0.0181)
FDI	−0.4620 ***	−0.5375 ***	−0.7083 ***	−0.7908 ***	−0.8044 ***	−0.9051 ***
	(0.1765)	(0.1361)	(0.1229)	(0.1010)	(0.1161)	(0.0979)
ER	−0.3619 ***	−0.1538 ***	−0.2044 ***	−0.0455 *	−0.1422 ***	−0.0316
	(0.0499)	(0.0385)	(0.0302)	(0.0248)	(0.0302)	(0.0255)
PDensity	0.1392	−0.1399	0.3088 ***	−0.0613	0.4794 ***	0.1360
	(0.1257)	(0.0969)	(0.1104)	(0.0907)	(0.1271)	(0.1072)
Human	0.0296 *	0.0330 ***	0.0444 ***	0.0449 ***	0.0296 ***	0.0339 ***
	(0.0157)	(0.0121)	(0.0110)	(0.0090)	(0.0090)	(0.0076)
Student	0.3443 ***	0.1853 **	0.3420 ***	0.1633 ***	0.3488 ***	0.2194 ***
	(0.1047)	(0.0808)	(0.0644)	(0.0529)	(0.0583)	(0.0492)
Digital	0.1120 ***	0.0354 *	0.1046 ***	0.0233 *	0.0934 ***	0.0243 *
	(0.0263)	(0.0203)	(0.0154)	(0.0126)	(0.0152)	(0.0128)
lnPatent	−0.0011	−0.0033	−0.0001	−0.0034 **	−0.0024	−0.0031 *
	(0.0033)	(0.0026)	(0.0021)	(0.0017)	(0.0019)	(0.0016)
Year/City fixed effect	Yes	Yes	Yes	Yes	Yes	Yes
R-squared	0.1565	0.5608	0.1672	0.5718	0.1758	0.5871
Number of cities	63	63	127	127	132	132

Note: Numbers in brackets are standard errors. Asterisks indicate the significance of the corresponding levels: *** (1%), ** (5%), and * (10%). The data were calculated by the authors using Stata 16.0.

**Table 7:** Empirical results of factor allocation efficiency analysis.

Variables	Capital Allocation Efficiency		Labor Allocation Efficiency	
	(1) PSM-Kernel	(2) Entropy Balancing	(3) PSM-Kernel	(4) Entropy Balancing
ORBArea × Post	−0.0537 ***	−0.0616 ***	−0.0153	−0.0041
	(0.0157)	(0.0150)	(0.0140)	(0.0132)
lnRGDP	0.1625 ***	0.1774 ***	0.2285 ***	0.2303 ***
	(0.0192)	(0.0190)	(0.0172)	(0.0167)
Gover	−0.8670 ***	−1.5625 ***	−1.8665 ***	−1.7452 ***
	(0.3053)	(0.3040)	(0.2726)	(0.2676)
Industry	0.1669 **	0.1098	−0.3710 ***	−0.2958 ***
	(0.0756)	(0.0698)	(0.0675)	(0.0614)
FDI	−3.8034 ***	−4.0893 ***	−0.0921	0.1918
	(0.3581)	(0.3425)	(0.3198)	(0.3015)
ER	0.7889 ***	0.7769 ***	−0.6582 ***	−0.5581 ***
	(0.0925)	(0.0966)	(0.0826)	(0.0850)
PDensity	−0.4775	−0.3370	−1.1739 ***	−0.9430 ***
	(0.3251)	(0.3888)	(0.2903)	(0.3422)
Human	0.0984 **	0.0867 **	0.1065 ***	0.0366
	(0.0391)	(0.0365)	(0.0349)	(0.0321)
Student	−1.0712 ***	−1.2351 ***	1.3467 ***	1.4035 ***
	(0.2175)	(0.2008)	(0.1942)	(0.1768)
City fixed effect	Yes	Yes	Yes	Yes
Year fixed effect	Yes	Yes	Yes	Yes
R-squared	0.3751	0.3990	0.3415	0.3545
Number of cities	116	119	116	119

Note: Numbers in brackets are standard errors. Asterisks indicate the significance of the corresponding levels: *** (1%), and ** (5%). The data were calculated by the authors using Stata 16.0.

**Table 8:** Empirical results of the industrial structure analysis.

Variables	Energy_Stru		lnFintech	
	(1) PSM-Kernel	(2) Entropy Balancing	(3) PSM-Kernel	(4) Entropy Balancing
ORBArea × Post	0.1219 ***	0.1517 ***	−0.1371 ***	−0.1470 ***
	(0.0235)	(0.0224)	(0.0331)	(0.0329)
lnRGDP	0.0517 *	0.0766 **	0.2008 ***	0.1698 ***
	(0.0303)	(0.0301)	(0.0427)	(0.0443)
Gover	0.2383	0.2826	2.0580 ***	2.0352 ***
	(0.4575)	(0.4528)	(0.6444)	(0.6661)
Industry	0.1141	0.1240	0.2238	0.1692
	(0.1160)	(0.1050)	(0.1633)	(0.1545)
FDI	1.9032 ***	1.8393 ***	2.5546 ***	2.8494 ***
	(0.5755)	(0.5613)	(0.8105)	(0.8259)
ER	1.3850 ***	1.2825 ***	0.1304	0.3662 *
	(0.1425)	(0.1471)	(0.2007)	(0.2164)
PDensity	0.2961	0.0880	0.2508	0.9717
	(0.5235)	(0.6219)	(0.7359)	(0.9144)
Human	0.0742	0.0723	0.0916	0.0260
	(0.0521)	(0.0441)	(0.0734)	(0.0648)
Student	−2.7052 ***	−2.6559 ***	−0.5695	−1.3112 ***
	(0.3040)	(0.2850)	(0.4281)	(0.4194)
City fixed effect	Yes	Yes	Yes	Yes
Year fixed effect	Yes	Yes	Yes	Yes
R-squared	0.1834	0.1937	0.9520	0.9528
Number of cities	127	132	127	132

Note: Numbers in brackets are standard errors. Asterisks indicate the significance of the corresponding levels: *** (1%), ** (5%), and * (10%). The data are calculated by the authors using Stata 16.0.

**Table 9:** Empirical results of the innovation effect analysis.

Variables	lnPatent		Tech_Fiscal		Digital	
	(1) PSM-Kernel	(2) Entropy Balancing	(3) PSM-Kernel	(4) Entropy Balancing	(5) PSM-Kernel	(6) Entropy Balancing
ORBArea×Post	0.0712	−0.0195	0.0582 ***	0.0565 ***	0.0223 ***	0.0287 ***
	(0.0524)	(0.0518)	(0.0194)	(0.0209)	(0.0072)	(0.0066)
lnRGDP	0.1915 ***	0.1962 ***	−0.0179	−0.0212	0.0171 *	0.0189 **
	(0.0677)	(0.0697)	(0.0251)	(0.0281)	(0.0093)	(0.0088)
Gover	1.0690	1.6742	1.9893 ***	2.2249 ***	0.8644 ***	0.7528 ***
	(1.0215)	(1.0482)	(0.3788)	(0.4230)	(0.1400)	(0.1328)
Industry	0.1557	−0.0685	0.1500	0.3074 ***	−0.0693 *	−0.0992 ***
	(0.2589)	(0.2431)	(0.0960)	(0.0981)	(0.0355)	(0.0308)
FDI	8.7154 ***	9.6763 ***	0.3079	0.2270	0.7252 ***	0.7035 ***
	(1.2848)	(1.2995)	(0.4764)	(0.5244)	(0.1761)	(0.1647)
ER	0.5097	0.5680 *	−0.1189	−0.0731	0.2439 ***	0.2647 ***
	(0.3181)	(0.3405)	(0.1179)	(0.1374)	(0.0436)	(0.0432)
PDensity	−3.4340 ***	−3.2898 **	−0.8963 **	−0.8844	0.3576 **	0.7189 ***
	(1.1666)	(1.4388)	(0.4325)	(0.5807)	(0.1599)	(0.1823)
Human	0.3572 ***	0.2826 ***	0.1320 ***	0.1356 ***	0.0453 ***	0.0324 **
	(0.1163)	(0.1020)	(0.0431)	(0.0412)	(0.0159)	(0.0129)
Student	−0.6654	−1.2685 *	0.1724	0.3640	−0.4664 ***	−0.3546 ***
	(0.6787)	(0.6599)	(0.2517)	(0.2663)	(0.0930)	(0.0836)
City fixed effect	Yes	Yes	Yes	Yes	Yes	Yes
Year fixed effect	Yes	Yes	Yes	Yes	Yes	Yes
R-squared	0.8900	0.8904	0.3019	0.2865	0.3134	0.3032
Number of cities	127	132	127	132	127	132

Note: Numbers in brackets are standard errors. Asterisks indicate the significance of the corresponding levels: *** (1%), ** (5%), and * (10%). The data were calculated by the authors using Stata 16.0.

**Table 10:** Empirical results of the pollution effect analysis.

Variables	PM2_5		Pollution_Water		Pollution_Gas	
	(1) PSM-Kernel	(2) Entropy Balancing	(3) PSM-Kernel	(4) Entropy Balancing	(5) PSM-Kernel	(6) Entropy Balancing
ORBArea × Post	−0.0475	−0.1818	−0.0314	−0.0622	−0.0612	0.0023
	(0.2452)	(0.2304)	(0.0386)	(0.0385)	(0.0449)	(0.0483)
lnRGDP	−0.9933 ***	−0.9442 ***	0.0199	0.0492	0.0403	0.2284 ***
	(0.3167)	(0.3099)	(0.0498)	(0.0518)	(0.0580)	(0.0650)
Gover	−14.6545 ***	−17.6681 ***	−0.3757	0.1815	1.9695 **	2.9853 ***
	(4.7792)	(4.6601)	(0.7518)	(0.7782)	(0.8755)	(0.9767)
Industry	2.8799 **	3.6535 ***	1.1773 ***	1.0952 ***	1.1398 ***	1.3961 ***
	(1.2112)	(1.0809)	(0.1905)	(0.1805)	(0.2219)	(0.2265)
FDI	3.6415	8.2122	−4.2298 ***	−4.9309 ***	−3.7499 ***	−3.6028 ***
	(6.0110)	(5.7774)	(0.9455)	(0.9647)	(1.1012)	(1.2109)
ER	8.6053 ***	9.2244 ***	1.3877 ***	1.1965 ***	1.4866 ***	0.9356 ***
	(1.4883)	(1.5138)	(0.2341)	(0.2528)	(0.2726)	(0.3173)
PDensity	−1.5079	−7.8833	−0.9476	−1.6464	−1.9423 *	−6.4445 ***
	(5.4579)	(6.3968)	(0.8585)	(1.0682)	(0.9998)	(1.3407)
Human	−1.5081 ***	−1.0603 **	0.0964	0.0795	0.0716	0.0725
	(0.5441)	(0.4535)	(0.0856)	(0.0757)	(0.0997)	(0.0951)
Student	0.1126	−2.4583	0.9429 *	1.1305 **	1.8159 ***	1.3941 **
	(3.1755)	(2.9337)	(0.4995)	(0.4899)	(0.5817)	(0.6149)
City fixed effect	Yes	Yes	Yes	Yes	Yes	Yes
Year fixed effect	No	Yes	No	Yes	No	Yes
R-squared	0.7785	0.7858	0.4190	0.4153	0.5799	0.5137
Number of cities	127	132	127	132	127	132

Note: Numbers in brackets are standard errors. Asterisks indicate the significance of the corresponding levels: *** (1%), ** (5%), and * (10%). The data were calculated by the authors using Stata 16.0.

**Table 11:** Empirical results on the moderating effect of the trade factors.

Variables	Moderator: Trade_Domestic		Moderator: Trade_Foreign	
	(1) SBM	(2) EBM	(3) SBM	(4) EBM
ORBArea × Post	−0.0156 *	−0.0041	−0.0171 ***	−0.0081 *
	(0.0081)	(0.0071)	(0.0053)	(0.0046)
ORBArea × Post × Moderator	−0.0290	−0.0605 ***	−0.0748 ***	−0.0379 *
	(0.0246)	(0.0214)	(0.0236)	(0.0206)
Moderator	−0.0101	−0.0092	−0.0467	−0.1042
	(0.0085)	(0.0074)	(0.0837)	(0.0731)
Controls	Yes	Yes	Yes	Yes
City/Year fixed effect	Yes	Yes	Yes	Yes
R-squared	0.1674	0.6238	0.1717	0.6232
Number of cities	132	132	132	132

Note: Numbers in brackets are standard errors. Asterisks indicate the significance of the corresponding levels: *** (1%), and * (10%). The DID regression was performed based on the entropy balancing matching in all columns. The data were calculated by the authors using Stata 16.0.

## Data Availability

The data presented in this study are available on request from the corresponding author.
